# SENOLYTIC EFFECTS ON COGNITION, MOBILITY, AND BIOMARKERS IN OLDER ADULTS WITH MCI AND SLOW GAIT: A PILOT STUDY

**DOI:** 10.1093/geroni/igae098.3709

**Published:** 2024-12-31

**Authors:** Courtney Millar, Ike Iloputaife, Kathryn Baldyga, Amani Norling, Afroditi Boulougoura, Theodoros Vichos, Tamara Tchkonia, Jim Kirkland

**Affiliations:** Hebrew SeniorLife, Roslindale, Massachusetts, United States; Hebrew SeniorLife, Roslindale, Massachusetts, United States; Hebrew SeniorLife, Roslindale, Massachusetts, United States; Hebrew SeniorLife, Marcus Institute of Aging, Harvard Medical Schools, Roslindale, Massachusetts, United States; BIDMC, Boston, Massachusetts, United States; BIDMC, Boston, Massachusetts, United States; Cedars-Sinai Medical Center, Los Angeles, California, United States; Cedars-Sinai Medical Center, Los Angeles, California, United States

## Abstract

One aim of this single-arm pilot study, called the STAMINA Study, evaluates the preliminary effects of two senolytic drugs, Dasatinib and Quercetin (DQ), on phenotypic and biological outcomes in older adults with slow gait speed and mild cognitive impairment. Twelve participants took six cycles of 100 mg of Dasatinib and 1250 mg of Quercetin for two days every two weeks over 12 weeks. Absolute changes in cognition and mobility, and percent changes in biomarkers were calculated. Spearman correlations between changes in phenotypic and biomarker outcomes were performed. Montreal Cognitive Assessment (MoCA) scores (mean change = 1.0 point, 95%CI: -0.7, 2.7) and stride length (mean change = 0.031 meters, 95% CI: -0.003, 0.066) tended to improve following DQ, without reaching statistical significance. However, MoCA scores did improve significantly by 2 points (95%CI: 0.1, 4.0) in those with the lowest baseline MoCA scores (18-25 points). Mean percent change in tumor necrosis factor-alpha (TNF-α), a key product of the senescence associated secretory phenotype (SASP), decreased following DQ (mean change = -3.0%, 95%CI: -13.0, 7.1), which was significant when compared to biomarker controls (p=0.04). Reduction in TNF-α was significantly correlated with increases in MoCA score (r=-0.65, p=0.02).This study suggests that intermittent DQ treatment may have functional benefits in older adults with slow gait speed and mild cognitive impairment. The observed reduction in TNF-α and its correlation with increases in MoCA scores suggests that DQ may improve cognition by modulating the SASP. However, these data are preliminary and must be interpreted with caution.

